# Supplementary material for: From sequence to enzyme mechanism using multi-label machine learning
Source: BMC Bioinformatics. 2014 May 19;15:150. doi: 10.1186/1471-2105-15-150 (PMC4229970; doi:10.1186/1471-2105-15-150)
Supplement: Additional file 2 — Java code of ml2db. Additional file ml2db_code.tar.gz contains the Java source code to run the multi-label machine learning experiments and save the results to database. The code’s Javadoc is included. [file 1471-2105-15-150-S2.zip › additional file 2/ml2db/utils/doc/index-files/index-22.html]

V-Index


JavaScript is disabled on your browser.


- Overview
- Package
- Class
- Use
- Tree
- Deprecated
- Index
- Help

- Prev Letter
- Next Letter

- Frames
- No Frames

- All Classes

A B C D E F G H I J K L M N O P Q R S T U V W X Y 


## V

VAL1 - Static variable in class uk.ac.standrews.utils.test.Data


VAL2 - Static variable in class uk.ac.standrews.utils.test.Data


VAL3 - Static variable in class uk.ac.standrews.utils.test.Data


VALA - Static variable in class uk.ac.standrews.utils.test.Data


VALB - Static variable in class uk.ac.standrews.utils.test.Data


valueExistsInTable(String, String) - Method in class uk.ac.standrews.utils.main.database.TableReader
:   Checks whether a certain row value exists in the given table.

values() - Static method in class uk.ac.standrews.utils.test.Data


values2() - Static method in class uk.ac.standrews.utils.test.Data


valuesExistsInTable(String, String, String, String) - Method in class uk.ac.standrews.utils.main.database.TableReader
:   Checks whether a row with the two given values exists in the given table.

valuesExistsInTable(String, String, String, String, String, String) - Method in class uk.ac.standrews.utils.main.database.TableReader


VARCHAR\_16\_SQL\_DATATYPE - Static variable in class uk.ac.standrews.utils.main.database.DbUtils


VARCHAR\_255\_SQL\_DATATYPE - Static variable in class uk.ac.standrews.utils.main.database.DbUtils


VARCHAR\_26\_SQL\_DATATYPE - Static variable in class uk.ac.standrews.utils.main.database.DbUtils


VARCHAR\_90\_SQL\_DATATYPE - Static variable in class uk.ac.standrews.utils.main.database.DbUtils


vectorFrequencies(Vector<Integer>) - Static method in class uk.ac.standrews.utils.main.stats.StatUtils
:   Records the freqencies with which the integers appear in the array

vectorFromList(ArrayList<String>) - Static method in class uk.ac.standrews.utils.main.collectionutils.ListUtils


VectorUtils - Class in uk.ac.standrews.utils.main.collectionutils


VectorUtils() - Constructor for class uk.ac.standrews.utils.main.collectionutils.VectorUtils

A B C D E F G H I J K L M N O P Q R S T U V W X Y

- Overview
- Package
- Class
- Use
- Tree
- Deprecated
- Index
- Help

- Prev Letter
- Next Letter

- Frames
- No Frames

- All Classes
